# Research on the Design of Assistant Basketball Teaching System Based on Big Data

**DOI:** 10.1155/2022/1499555

**Published:** 2022-05-06

**Authors:** Lin Wang, Xiaoyun Wei, Guoliang Yuan

**Affiliations:** ^1^Department of Physical Education, North China University of Science and Technology, Hebei, China; ^2^College of Physical Education, Hengshui University, Hebei, China

## Abstract

Due to the limitations of the current teaching resources searched by the system, it is difficult to achieve good results in basketball teaching. For this reason, an auxiliary basketball teaching system based on big data is designed. On the basis of clarifying the basic principles of teaching system design, JSP (Java Server Pages) dynamic web page back-end technology and Tomcat 7.0 server are used as the system support structure. Take the big data environment as the source of teaching resources and match them to different databases according to the characteristics of the resources. And combined with real-time evaluation to achieve the assessment of teaching effects and the management of teaching resources, the test results show that the average score of the basketball technical action test under the guidance of this system is higher than 7.5 points when the experimental subjects have the same level of basketball skills. In the paired sample *t*-test and the independent sample *t*-test of the performance level test scores *P*=0.001 < 0.05, there is a significant difference. It shows that the system can effectively improve the teaching effect of basketball and improve students' basketball test scores.

## 1. Introduction

With the increasing maturity of Internet technology, computer technology, and information technology, distance education and education system based on the network have made great development and progress. Moreover, at this stage, multimedia technology has fully integrated network communication technology, and teaching work is carried out more in the network conditions, which has completely changed the teaching mode. Due to the shortcomings of the traditional model limited by space and time conditions, the network teaching model has the characteristics of autonomy and interaction, which makes students vulnerable to influence in the learning process. The communication between students and teachers is easier and closer. The defect that the traditional single auxiliary teaching mode cannot quickly feed back all kinds of information has also been solved by the network teaching mode [1–3]. As a bilateral teaching activity, physical education teaching embodies a very significant practicality in the process. During the lecture, teachers should explain and demonstrate at the same time and deepen students' understanding through visual teaching so as to make students have higher and more comprehensive quality and adapt to more advanced physical training and teaching in the future. Sports network teaching system is based on campus network, Internet, and other hardware and gives full play to the effectiveness of the network. In a real sense, it widely promotes and uses the new teaching method of network teaching, which is a problem that the industry needs to pay attention to and solve at this stage [4–6].

The main goal of the basketball auxiliary teaching system is to establish an auxiliary physical education system with the help of various types of networks such as campus networks and the Internet. And based on this platform, teachers can announce the specific activities of basketball teaching, disseminate various sports information and news at the same time, interact with students on the platform, and monitor, test, and collect various parameters related to students' physical fitness so that students can develop good and healthy exercise habits [7]. With the help of this system, students can choose physical education courses consistent with their own conditions according to their actual physical condition [8] and independently design and formulate exercise plans to make their living habits and life attitudes more healthy. At the same time, teachers can publish the specific teaching content of each class on this platform so as to make students more fully prepared. Students can also use this platform to select courses so as to make the course selection more purposeful and prevent the selected courses from not suitable for themselves, which can more effectively improve students' physical and physical quality [9, 10]. The teaching efficiency of physical education teachers can also be improved, and it will be more convenient for teachers to organize sports activities so as to make this platform play the function of auxiliary teaching. Network teaching mode has its own characteristics and advantages, which can not only fully mobilize students' learning enthusiasm but also completely change the disadvantages of the traditional mode focusing on classroom and teacher, which can promote the diversification of teaching forms and the interactivity and expansibility of teaching. Therefore, the teaching mode has been reformed and innovated.

In related research, some scholars have designed a basketball tactical awareness auxiliary training system based on multimedia technology. Based on professional theory and technology, we designed a multimedia auxiliary training system. The test method of the system can objectively reflect the students' basketball tactical awareness level. However, there is less resource information in basketball tactics analysis, which is not obvious for long-term basketball training [11]. Some scholars have proposed a basketball teaching and training system that combines multimedia interactive mode and virtual reality technology. This method establishes a basketball movement model and an elastic deformation model through a mathematical model. Then through the virtual reality model, the basketball players are modeled geometrically and kinematically to capture the information generated in the basketball tactical training. The multimedia signal processing method is used to process the information, and the basketball observation and feedback based on the multimedia interactive model and virtual reality technology is constructed. However, the system's classification of basketball training resources is not accurate enough and needs to be further improved [12].

Based on this, this article designs an auxiliary basketball teaching system based on big data. In the hardware part, the browser end environment and the server port software and hardware environment are designed. In the software part, the technical architecture, web page architecture, system modules, core functions, and teaching resource management methods are designed. The practical application effect in teaching is verified by application test.

## 2. System Design Principles

Auxiliary teaching system is the product of modern information technology, and its development and design must meet the basic norms and requirements of current information technology [13–15]. Therefore, before designing the auxiliary teaching system, we must determine the guiding principles to promote the product of the system in the information age.

### 2.1. Principle of Availability and Ease of Use

The availability and ease of use of the system is the primary principle of the design of basketball auxiliary teaching system. The development and design of the system should ensure the smooth use in the teaching process and avoid the situation of being unable to serve as much as possible. Ease of use is an important guarantee to ensure the long-term sustainable use of users. Therefore, in the development process, it is necessary to design the user's operation experience from multiple angles in combination with the needs of different users so as to continuously improve the ease of use of the system such as providing system operation-friendly prompt, reducing user production, and economic input.

### 2.2. Principle of Practicality and Stability

The system design should fully consider the interactive process of basketball teachers' teaching and students' learning and combine the existing teaching management business process to minimize the redundancy of system data. At the same time, the design of the system function and interface should not only comply with the specifications of the auxiliary education system but also make the user's operation process concise and master the operation of the system quickly. In addition, maintaining the stability of the auxiliary teaching system is the first criterion. The stability of the system will directly affect the user's operation experience. If there are many failures or system crashes in the use process, it will be difficult to achieve the original intention of improving the teaching quality.

### 2.3. Maintainability and Scalability Principle

The continuous renewal of information technology, the advancement of basketball teaching, and the changes of students' characteristics require the continuous development and reform of the development and design of this system to meet the needs of teaching so as to meet the needs of teaching students in accordance with their aptitude. Therefore, based on the real-time change of basketball teaching needs, the system design in different development versions should fully consider the maintainability and scalability of the system rather than a one-time project. Software maintainability can be measured by reliability, testability, understandability, modifiability, and so on.

### 2.4. Safety Principle

Whether the system is safe or not is an important index to evaluate the excellence or inferiority of a software program. In the era of modern information technology, B/S architecture is very vulnerable to attack due to open-source sharing. Therefore, system security must be given priority in system development. For example, the security of the system is ensured by filtering and controlling the IP of system visitors, controlling the user's use authority, limiting the user's input characters, etc. In addition, when building the system, reduce system vulnerabilities as much as possible and take necessary security measures to improve the reliability and security of system operation and prevent system data loss, destruction, and theft.

## 3. System Development Environment Design

System development environment (SDE) is an application software used in response to the engineering development and maintenance of application software and system software based on basic hardware and digital software, which is called SDE. SDE is composed of software tools and environment integration mechanism [16, 17]. Software tools are used to support software R & D processes, tasks, and activities. Environment integration mechanism provides unified platform support for tool integration and software development, maintenance, and management. According to the system function and system hardware requirements, this study standardizes the required environment for system development, mainly from the hardware environment and software environment, including the browser end and the server end.

### 3.1. Browser Environment Design

In order to meet the accessibility requirements of different users on the browser side [18, 19], the configuration requirements of the equipment during system development shall be minimized as far as possible so as to meet most browser in the market. The system uses JSP (Java Server Pages) dynamic web page back-end technology to contact the browser-side HTML web page system operation interface and server database. JSP is not only a java server page but also a dynamic web page technical standard. Its function library is powerful, the original code is open to the society, and JSP dynamic web pages on the market are supported by most browsers. Therefore, the system does not limit the user's operating environment on the browser side but improves the browser-side environment. As long as the user side has a computer, Internet equipment, e-mail browser, and web browser, the system can be used. The browser system requires that the application software be Internet Explorer, 360, Firefox, QQ, and other browser. The operating systems are Windows, IOS, Android, etc. The operating equipment is computer, pad, mobile phone, etc.

### 3.2. Server Software and Hardware Environment Design

The server-side system architecture uses Tomcat 7.0 server and the built-in SQL SERVER2019 version of MyEclipse 2019 to establish the system database, which is set up on the Microsoft Windows7 computer host, with new FTP server, web server, mail server, and other subsystems. The requirements of the server side for the system are as follows:

  Network programming language: PHP, ASP.NET, JSP
  Protocol: TCP/IP
  Website system/database: Apache Web Server, SQL SERVER2019
  Operating system: Microsoft Windows 7

The specific system development environment is shown in Table 1.

On this basis, the auxiliary basketball teaching system is designed.

## 4. System Software Design

### 4.1. Framework Design

#### 4.1.1. Technical Framework Design

**Figure 1:**
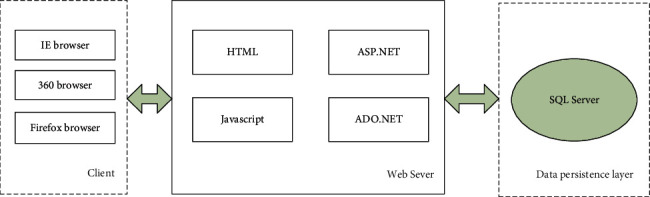
Framework of auxiliary basketball teaching system.

The system architecture design uses B/S architecture [20] and ASP.Net technology to develop and design the auxiliary teaching system, which is characterized by simple deployment and convenient maintenance. The web server is deployed by IIS server, and the database is managed by sql2015 database management system. The UI, business logic handler, and data access logic are all deployed by the web server. The data access and control program uses PHP technology to connect and access to SQL Server database. The user interface uses HTML, Java, and other technologies to design and implement the UI interface. The UI interface interaction is realized by using the relevant components of ASP.Net. The data structure, business thinking logic, and view presentation under this system are relatively separate and specific, which is more conducive to the maintainability and expansibility of the system. The overall architecture of the system is shown in Figure 1.

The overall reality of the system architecture is combined with the application mode with strong practicability. As shown in Figure 1, the system architecture follows the J2EE development standard framework and designs three specific layers including presentation layer, business layer, and data persistence layer. In practical application, combined with the automatic code generation tool, it fully supports the whole system architecture and can also generate a large number of repetitive basic codes in line with the overall architecture in a short time so that the actual development of the auxiliary teaching system focuses on the development and design of business logic, which effectively reduces the resource consumption of some links during system development. The quality and construction efficiency of the system have been greatly improved. The system architecture is described as follows:

1. The interface layer interface mainly provides an interactive display interface for the teaching system and users. It is located at the customer end of the system software to preliminarily verify the data on the display interface. When in use, the service request is sent from the web browser and then received by the web server. The request information sent by the user is encapsulated internally, processed by the web server, and then, the portal is displayed through the user's browser in a specified way to separate the page display from the data content. It is an international standard mechanism in modern information technology. It can form a consistent abnormal situation treatment, which is flexible, simple, effective, and convenient for function expansion and maintenance.
2. The business layer converts the outgoing data structure of the interface layer into the data of the business layer and then processes it. It is responsible for the logical thinking processing of various business work. After the system runs, it first accepts the user's request, obtains the user data from the request data, calculates and processes it according to specific rules, carries out interactive processing through components and services, and finally uploads the processing results to the server, which encapsulates the processed results again and returns them to the client.
3. The data persistence layer is a relatively independent field to realize data persistence to greatly reduce the time of manual development using JDBC (Java Data Base Connectivity) and SQL to process data. This layer is mainly responsible for data interaction with the underlying database system, including the logical processing of system data; accepting the request of the web server; completing the query, modification, and other operations of the database through the internal interface; and transmitting all the processed results to the server.

#### 4.1.2. Network Architecture Design

**Figure 2:**
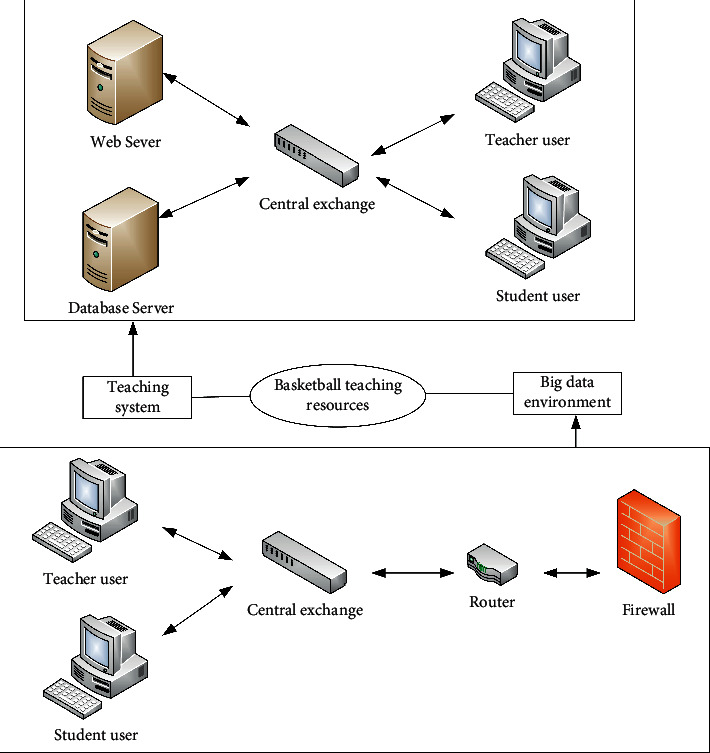
Network architecture of basketball auxiliary teaching system.

Network architecture is a blueprint that provides an architecture and technical basis for the design, construction, and management of a communication network. When designing the network architecture, the auxiliary teaching system combines the actual needs of basketball teaching course construction and can show very strong security and convenience so that users can carry out auxiliary learning on the school external network or internal LAN at any time according to their actual situation. The network architecture of the system is shown in Figure 2.

Network architecture is a prerequisite for the operation of the network system. As shown in Figure 2, the network setting of the system can collect resources that meet the needs of teaching application in the big data environment in the LAN, which is not only consistent with the characteristics of basketball teaching but also meets the environmental conditions of existing basketball teaching. From the internal architecture, the server is divided into database server and web server. The internal communication carries out the flow of information through the network switch. The collection of basketball auxiliary teaching resources in the big data environment needs to enter the teaching system through firewall, VPN, and other measures and settings, including switches, routers, and other network equipment. In this data flow process, all comply with HTTP network protocol.

### 4.2. System Module Design

According to the regional division of system functions, the basketball auxiliary teaching system is set as 7 functional modules such as system management module, data management module, and ability evaluation module as shown in Table 2. The design of the system module will provide an important design basis for the development of the system home page.

The modules in Table 2 are independent and interrelated. While undertaking their own system tasks, they also support each other with the functions of other modules. On the whole, the system consists of seven modules, which constitute an organic whole of basketball-assisted teaching. It conforms to the characteristics of basketball teaching and plays an important auxiliary role in teaching activities. Specifically, each functional module of the system has its own functional characteristics. The system management module establishes the operation mechanism of the whole system and standardizes the authority setting during the operation of the system, which is an important guarantee for the normal operation of the system. It is mainly responsible for the security and stability of the system in order to provide sustainable services for system users. The data management module is the initial function of basketball auxiliary teaching, which provides demand information for the application of other functions. It is closely related to the ease of use of the system, and its basic data are important basis for the redevelopment of the system. The ability evaluation module innovates the application of auxiliary teaching in sports technical teaching and enriches the functional design of auxiliary teaching system. The assessment management module is mainly used to manage the assessment scheme, formulate the assessment content, manage the score evaluation, and manage the examination process and the publication, ranking, and archiving of scores. The homework management module is mainly used for teaching homework management, including teachers issuing homework after class, correcting homework, and students submitting homework answers. The interactive management module builds an interactive platform for teachers and students to facilitate timely communication and solve students' questions in the process of teaching and practice in real time. The teaching resource management module is to build a basketball teaching video resource database to facilitate students' resource sharing and learning anytime and anywhere.

### 4.3. Core Function Design

#### 4.3.1. Design of Technical Assessment Based on Real-Time Assessment

The design is completed in VS 2019 environment. OpenGL port is used as the external access port for graphics processing and control, and the interaction with 3D design is realized at the same time. According to the multiple practice characteristics of basketball learning process, the design requires students to practice many times before, during, and after class in order to meet the assessment standard of technical action. By inputting the basketball standard assessment technology demonstration video, the upload channel of students' independent assessment video is designed to realize the interaction between demonstration and assessment files. Therefore, this paper first constructs the assessment framework *K* and describes all possible test results within the framework. If the appraisal result meets the requirements, the result is *k*_1_, the local error result is *k*_2_, the complete error result is *k*_3_, and the uncertain result is set *k*, then

(1) $$\begin{array}{c} K=\left\{k_{1},k_{2},k_{3},\kappa \right\}. \end{array}$$

Next, set its allocation weight. Within the appraisal framework *K*, the basic allocation weight is *P*, and the conditions *P*(*ϕ*)=0 are ∑_*k*⊆*K*_*P*(*k*)=1 met. Then, *P*(*k*) can represent the basic appraisal index and calculate the distribution weight of all results within the appraisal framework. The result is

(2) $$\begin{array}{c} P\left(k_{i}\right)=\lambda_{i}\gamma_{ij},\\ P\left(K\right)=1-\lambda_{i}, \end{array}$$

wherein *P*(*k*_*i*_) represents the distribution weight of the *i* assessment result to the test target, *P*(*K*) represents the distribution weight of the *i* uncertain test result to the test target, *λ*_*i*_ represents the reliability coefficient of the *i* test result, and *γ*_*ij*_ represents the membership of the *i* assessment result to the wrong result *j*.

After the assigned weight of the test results is obtained, the evaluation results can be fused. The calculation method is

(3) $$\begin{array}{c} P{}^{\prime }\left(k_{i}\right)=\frac{\sum_{k\in K}P\left(k_{i}\right)}{P\left(K\right)}. \end{array}$$

Finally, according to the fusion results, the decision is made whether the test results are abnormal. When *P*′(*k*_*i*_)=max{*P*(*k*_*i*_), *k*_*i*_ ∈ *K*}, the result is excellent; when *P*′(*k*_*i*_) ≥ *P*(*K*), the result is qualified; when *P*′(*k*_*i*_)=0, the result is unqualified.

On this basis, the system function class design mainly includes loading files, resource upload, data files, real-time comparison, and evaluation between teachers and students; through the core control class function design, we can realize the real-time evaluation of students' ability in basketball teaching process, the evaluation and archiving of technical evaluation data, etc.

#### 4.3.2. Teaching Resource Management Design

Teaching resources refer to all human and nonhuman resources that can be used by learners in the learning process, including information, materials, equipment, personnel, and places. The processing of teaching resources this time is mainly for the relevant data and image materials for guiding the training of students. The teaching resource management module mainly refers to the management module that provides teaching resource operation for the role of using the educational administration management of the system. We systematically classify various teaching resources in teachers' basketball teaching. Through the report and analysis system provided by the system, the educational administration department will be able to accurately grasp the distribution of various teaching resources in the school and take this as the basis to make a reasonable plan for the construction of teaching resources in the future.

**Figure 3:**
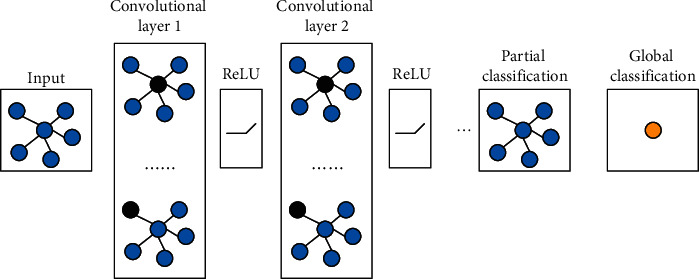
Graph neural network framework.

First, it is necessary to extract the characteristics of basketball actions, and on this basis, it is possible to efficiently organize relevant basketball teaching resources. The graph convolutional neural network is used here. Since the image is a two-dimensional data structure, the main idea of CNN is to design a suitable convolution kernel to form a sliding window model on the image and then perform repeated translation of the sliding window convolution operation to extract features in image data. For the behavior recognition algorithm in this paper, the process belongs to a global classification task. The framework of graph neural network is shown in Figure 3.

The image convolutional neural network mainly uses the spatial convolution method when extracting the features of the key points of the human skeleton. The spatial convolution method refers to the decomposition of a node into two processes: message transfer and status update operations. Taking the two-dimensional convolution of a common image as an example, the convolution output for a certain position *x* can be written as

(4) $$\begin{array}{c} f\left(x\right)=\sum_{a=1}^{k}cf_{i}\cdot p\left(a,w\right). \end{array}$$

In formula (4), *f*_*i*_ is the feature map of input channel *c*, *k* is the number of convolutions, *p*(*a*, *w*) is the sampling function, and *x* pixel is taken as the pixel center. In basketball action images, the neighbor pixel geometry is defined as follows:

(5) $$\begin{array}{c} Q\left(v_{ce}\right)=v_{ci}|d\left(v_{ci},v_{ce}\right). \end{array}$$

In formula (5), *d*(*v*_*ci*_, *v*_*ce*_) is the shortest distance from *v*_*ci*_ to *v*_*ce*_, so the adopted function can be written as *p*(*v*_*ci*_, *v*_*ce*_). Analogous to 2D convolution, the neighbor pixels obtained by the sampling function in the graph are divided into different subsets, and each subset has a digital label. Mapping a neighbor node to the corresponding subset label, the collection weight of the basketball action feature is as follows:

(6) $$\begin{array}{c} \omega =\sum_{o\in Q\left(v_{ce}\right)}of\left(x\right)\cdot p\left(v_{ci},v_{ce}\right). \end{array}$$

Let the system resource distribution be a directed graph *I*(*D*, *L*), where *D* represents the vertex of the resource distribution database and thus the corresponding node, *L* represents the resource characteristics of different databases, and the set of database and resource characteristics is represented as

(7) $$\begin{array}{c} D=\left\{d_{1},d_{2},\ldots ,d_{n}\right\},\\ L=\left\{l_{1},l_{2},\ldots l_{m}\right\}, \end{array}$$

where *n* represents the number of databases in the system, *m* represents the number of corresponding database resource characteristics, and the variable parameter is *λ*_*a*,*b*_, which is defined as

(8) $$\begin{array}{c} \lambda_{a,b}=\left\{\begin{array}{cc} 1, & \text{The same characteristics exist between databases }a\text{ and }b,\\ 0, & \text{The same characteristics do not exist between databases }a\text{ and }b. \end{array}\right) \end{array}$$

Similarity *D*(*l*) and relevance *F*(*l*) of resource characteristics are expressed as

(9) $$\begin{array}{c} D\left(l\right)=\sum_{a=1}^{n}\sum_{b=1}^{n}\min d_{a,b}\lambda_{a,b},\\ F\left(l\right)=\sum_{a=1}^{n}\sum_{b=1}^{n}\min f_{a,b}\lambda_{a,b}, \end{array}$$

where *a*, *b*=1,2,…, *n*, *d*_*a*,*b*_, and *f*_*a*,*b*_, respectively, represent the average degree and average correlation of resource characteristics between databases *a* and *b*.

According to the calculation results, the teaching resources obtained from the big data environment are allocated to the corresponding database.

### 4.4. System Database Design

Database design is the foundation of system development. Database design includes three parts: conceptual model design, logical model design, and physical implementation scheme design. It plays an important role in information system design and is an important guarantee to ensure the stable operation and sustainable development of the system. During system development, all business data are stored in the database. In order to ensure the normal operation of the system, it is required that the database structure of the system is reasonable and the data relational table runs efficiently. At the same time, the database design with clear logic, on the one hand, can save storage space, on the other hand, can greatly simplify the programming code, and can also facilitate the programmer's daily operation of adding, subtracting, checking, and repairing the database. In the development of the auxiliary teaching system, this study designs the database, strictly follows the database design steps, analyzes the needs of basketball auxiliary teaching, and then extracts the data and makes in-depth investigation and analysis. Finally, through the design of abstract conceptual model and logical model, this paper constructs the database of basketball auxiliary teaching system, which is based on big data resource *R*(*l*).

(10) $$\begin{array}{c} R\left(l\right)=\sum_{a=1}^{n}\sum_{b=1}^{n}B{}^{\prime }\lambda_{a,b}D\left(l\right)=B{}^{\prime }D\left(l\right)+F\left(l\right), \end{array}$$

where *B*′ represents the correlation coefficient between big data resources and teaching tasks so as to ensure the security of system resources.

So far, the design of the auxiliary basketball teaching system based on big data has been completed. The system software is designed on the basis of the hardware browser end environment design and the server port environment. The system software runs on the basis of the hardware environment. The hardware environment designed this time has strong operation protection performance, can support the use of teachers and student users, and can quickly search and store teaching resources. In the software part, it deals with the basic system web page structure, system module design, and mainly uses big data to design the classification and management process of teaching resources. This solves the problem of limited teaching resources and improves the performance of basketball teaching.

## 5. Application Test

### 5.1. Research Object

This subject takes the effect of basketball skill improvement applied by auxiliary basketball teaching system in basketball teaching as the research object. Through the basketball teaching experiment of 40 students in the basketball special elective class of the Department of Physical Education of a grade in the Physical Education College of a university, this paper analyzes the application effect of the auxiliary teaching system designed in this paper; The quasiexperimental design of equal time samples was carried out. In order to ensure the authenticity and feasibility of the experiment and the effectiveness and scientificity of the results, the experimental information is strictly kept confidential to ensure that the experimental objects participating in the teaching experiment do not know about the experiment and minimize the influencing factors of the experiment. 40 students were divided into control group and experimental group. The experimental group used the auxiliary teaching system designed in this paper for basketball teaching, and the control group used conventional basketball teaching. Other conditions were the same. Before the experiment, ensure that there is no difference in the basketball skill level of the subjects. After the experiment, test the basketball skill level of the two groups of subjects, compare and analyze the test results, and draw a conclusion.

### 5.2. Supporting Conditions for This Study

The University carrying out the experiment is a sports college with good school running qualification. Its students have won the championship in the competition for many times. At the same time, the teachers of the basketball teaching and Research Office of the College of Physical Education of the University led the school basketball team to obtain excellent results many times in the competition. The teaching staff of basketball specialty is strong, and the teaching facilities are perfect, which laid the foundation for the effective development of this study. On this basis, the experimental study takes four basketball teachers from the basketball teaching and Research Department of the College of Physical Education of the University as the evaluators. Before and after the experiment, the basketball skill level of the experimental subjects is evaluated. The basketball skill test indicators are front changing dribble, crotch dribble, back dribble, three-step layup, and fixed-point shooting.

### 5.3. Skill Test and Grouping of Subjects before Experiment

#### 5.3.1. Grouping Test Criteria

Before the experiment, four professional teachers from the basketball teaching and research department were invited to conduct the pretest on 40 randomly selected students from the basketball special elective class. The technical action forward change dribble, crotch dribble, back dribble, three-step layup, and fixed-point shooting were taken as the test contents, and the scores were scored and recorded according to the standard degree of students' action completion. The full score of the technical and tactical evaluation of a single action was 10 points. There are 8 items in total, and the total score of each student is 80.

#### 5.3.2. Grouping Process and Inspection

*(1) Grouping Process of Subjects before Experiment*. According to the evaluation results of 40 experimental subjects by four basketball teachers, cross-grouping is carried out (that is, according to the evaluation results of 40 students, the scores are ranked from high to low as 1 to 40, and the students with adjacent scores are paired from 1. After all pairing is completed, one of the paired students is assigned to the experimental group, and the other is the control group). They were divided into experimental group and control group. The students in the experimental group and control group were numbered 1∼20 in turn. According to the evaluation results of each student by four teachers (the full score of each student's single test is 10 points, a total of 8 test contents, and the total score is 80 points), the average value of the overall single scores of the two groups of students is obtained as shown in Tables 3 and 4.

*(2) T-Test*. According to the average student test scores in Table 3, the homogeneity of the scores of the two groups of students is tested by SPSS 22.0. The results are shown in Table 4.

According to Table 4, *P*=0.727 < 0.05 has the characteristics of no significant difference, indicating that before the experiment, there is no difference in the basketball skill level between the control group and the experimental group, which meets the conditions of homogeneity test. Therefore, the basketball skill level of the experimental subjects is equivalent and meets the experimental requirements, so the experimental research can be carried out further.

### 5.4. Teaching Process of Experimental Objects

On the basis of following the rules of conventional basketball teaching courses, this experimental teaching ensures that the teaching contents of the experimental group are consistent with those of the control group. The technical action teaching contents are front changing dribble, crotch dribble, back dribble, three-step layup, and fixed-point shooting. At the same time, in order to better ensure the reliability of the experimental effect, the experimental group and the control group were taught by the same basketball teacher. The operations of the experimental group and the control group are as follows.

#### 5.4.1. Basketball Teaching Process of Experimental Group

In the experimental group, the student number, name, gender, and class information of the students are input into the design system in advance before the experiment, and the class name is named the experimental group.

Course Preparation: at the end of the experiment, the teacher will notify the students through the system designed in this paper before each class session (each student may be notified) to encourage them to preview the course in advance. During the preview session, the students may participate in an auxiliary interactive discussion activity, and the teacher may record key questions discussed in the class for answers. After each class, the teacher will arrange assignments related to the content of the course to allow the students to test themselves. Most of these studies focused on video assignments, such as the three-step layup process of the explanation. Let the students watch the video of the three-step layup and upload it to the operating area of the system for the teachers to check their homework after class. When students submit their homework, teachers can clearly understand the dynamics of students' assisted learning through the system designed in this paper so as to form active and effective supervision over students' learning.

The beginning of the course: at the beginning of the course, in the first 10 minutes, the teacher organized the students of the experimental group to uniformly watch the resources in the teaching system by using the LED electronic display screen of the indoor basketball court of the comprehensive training hall of the College of Physical Education of Zhengzhou University. After watching, the teacher organized the students to discuss and answer questions on the spot and answered the concentrated questions in the preclass preview and classroom discussion. The discussion time is generally controlled within 10 minutes. After watching the basketball teaching resources in the design system, and after the discussion and Q & A, the teachers organize the students to start group practice, follow the basic rules of basketball course, and reasonably arrange the training and rest time. Except for the viewing of the teaching resources and discussion and Q & A activities in the system at the beginning of the course, other teaching arrangements are consistent with those in the control group.

The end of the course: the teacher summarizes and comments on the training effect and precautions of this course, summarizes the teaching content of this course, and arranges the training homework after class, and the homework is submitted through the design system of this paper.

#### 5.4.2. Control Group Basketball Teaching Process

The teaching process of the control group followed the conventional basketball teaching mode. The teachers did not organize the students to study before class, and the students freely arranged to study or not study, without the help of the system designed in this paper. After the teacher explained and demonstrated the course, the students were organized to practice in groups, and the end of the course was summarized and fed back so as to timely adjust the deficiencies in the teaching. Except for preclass preparation and 20 minutes' viewing before class, the teaching resources, discussion, and Q & A of the system designed in this paper are consistent with the experimental group.

### 5.5. Control of Irrelevant Variables in the Experiment

In order to minimize the experimental error, the original teaching plan should not be disturbed in the teaching process of the experimental group and the control group. Except for the students in the experimental class, they are encouraged to study the teaching resources of the design system independently before class, watch the teaching resources of the design system in class, and submit the homework through the design system after class, and the other conditions are consistent with the control group. At the same time, in order to reduce the experimental errors caused by teachers' differences, the experimental group and the control group were both taught by the same teacher, and the four teachers in charge of the test did not know that the test group or the control group was tested before and after the experiment so as to ensure the maximum fairness and authenticity of the test results. The Hawthorne effect refers to the methodological artificial effect brought about by the research subjects realizing that they are being studied in behavioral field experiments. In order to avoid the Hawthorne effect, we should keep the information of the experiment strictly, make sure that the subjects don't know the experiment, avoid the abnormal state of mind, and improve the reliability of the conclusion. At the same time, we should strictly control the students' autonomous practice after class and try our best to ensure the influence of individual's autonomous training on the experimental results. Finally, the 4 selected teachers and the students of each skill assessment standards are consistent with the results of the average scores.

### 5.6. Statistical Method of Test Results

After the end of this teaching experiment, four professional teachers of basketball teaching and research room were organized to test the basketball skill level of the experimental group and the control group. The test contents were the same as before the experiment (the technical action test contents were front changing dribble, crotch dribble, back dribble, three-step layup, and fixed-point shooting). Mathematical statistics this study uses is SPSS 22.0, mainly using independent sample *t*-test and paired sample *t*-test to make scientific statistical analysis of relevant data in the process of experimental research so as to ensure the scientificity and effectiveness of experimental results. Independent sample *t*-test is used to test the comparison of the mean test scores of the two groups of experimental groups in the experiment or after the experiment and to test whether there are differences in the mean between different groups. Paired sample *t*-test is used to test whether the average scores of the same group of experimental groups are different after experimental operation and before experiment.

### 5.7. Test Results and Analysis

#### 5.7.1. Comparison of Skill Levels before and after the Experiment

Before and after the experiment, the test scores of the experimental group were consistent with the *t*-test. After the teaching experiment, the average scores of four professional teachers in the basketball teaching and research room for each group of students in each test item are taken as the final scores of each student. The test items were the same as before the experiment (the technical action test contents include forward changing dribble, crotch dribble, back dribble, three-step layup, and fixed-point shooting). The full score of the single test index is 10 points, and the total score is 80 points. The average test scores of the two groups of students after the experiment are shown in Table 5.

It can be seen from Table 5 that after the experiment, the average scores of various test indexes of the experimental group are higher than those of the control group. And the average scores of the action test in the experimental group were all higher than 7.58. The average scores of the control group's action tests were all lower than 7.56 because this article takes the big data environment as the source of teaching resources and uses graph neural network to calculate the similarity between the characteristics of teaching resources and learners, which improves the search performance of teaching resources. Comparing the skill index test scores of the experimental group and the control group before the experiment (Table 3), it can be found that the test scores of the experimental group and the control group after the experiment are higher than those before the experiment.

Paired sample *t*-test was conducted for the test results of the experimental group before and after the experiment. The results are shown in Table 6.

From Table 6, *P*=0.001 < 0.05, with a very significant difference level, indicating that there is a great difference in the basketball technical action test results of the experimental group before and after the experiment, and the average basketball skill level of the experimental group after the experiment is higher than that before the experiment. Therefore, the basketball technical action level of the experimental group is significantly improved through this experiment.

#### 5.7.2. Paired Sample *T*-Test of Test Results of the Control Group before and after the Experiment

The average basketball skill level test scores of the control group before and after the experiment were sorted out, and SPSS 22.0 was used to conduct independent sample *t*-test on the data. The results are shown in Table 7.

According to Table 7, *P*=0.001 < 0.05, with a very significant difference, indicating that there is a great difference in the basketball technical action test results of the control group before and after the experiment, and the test mean after the experiment is greater than that before the experiment, that is, the basketball skill level after the experiment is higher than that before the experiment. Therefore, through this experiment, the basketball technical action level of the control group is significantly improved.

#### 5.7.3. Comparison of Skill Level between Experimental Group and Control Group after Experiment

SPSS 22.0 is used to conduct independent sample *t*-test on the basketball technical action test scores of the experimental group and the control group after the experiment, and the results are as follows.

According to Table 8, the independent sample *t*-test *P*=0.03 < 0.05, with a significant level, indicating that there is a significant difference in the scores of the two groups of students. Through the analysis of test data, it can be seen that there is a great difference in the basketball technical action level between the experimental group and the control group after the experiment, and the average score of the experimental group is greater than that of the control group. Therefore, after the experiment, the basketball technical action level of the students in the experimental group was higher than that of the control students.

To sum up, it can be concluded that through this experiment, the basketball skill level of the students in the experimental group is higher than that of the control group. This experiment not only improves the basketball skill level of the experimental objects but also proves that the performance of the experimental group using the basketball auxiliary teaching system designed in this paper is higher than the overall skill level of the control group using conventional teaching methods.

## 6. Conclusion

With the in-depth study of education informatization, smart classrooms have become one of the hot topics in current research. In this paper, an auxiliary basketball teaching system based on big data has been designed. The main conclusions can be summarized as follows: (1) the average score in the basketball technical action test is higher than 7.5 points. This system can effectively improve students' basketball test scores. (2) In the paired sample *t*-test and the independent sample *t*-test of the performance level test scores *P*=0.001 < 0.05, there is a significant difference. It shows that the system can effectively improve the teaching effect of basketball and improve students' basketball test scores. (3) The system can improve students' enthusiasm for learning, reduce the workload of teachers, and has high practical application value. In the future work, it is necessary to carry out research on the animation generation of basketball technical courseware to improve the teaching effect.

## Figures and Tables

**Table 1:** Development environment of basketball auxiliary teaching system.

Environmental sequence	Development environment requirements		Remarks
	Software	Hardware	
Web server	Apache Tomcat 7.0.0	Intel Core i5-7500 3.40 GHz, 8 GB memory, and 1TB hard disk	Application server of auxiliary teaching system
Database server	MySQL 2017	Intel Core i5-7500 3.40 GHz, 8GB memory, and 1TB hard disk	
System development platform	MyEclipse		
System development environment	Java		

**Table 2:** Module setting of basketball auxiliary teaching system.

System	System module	Module function setting
Basketball assistant teaching system	System management module	Login, password service, rights management, data backup, and data recovery
	Data management module	Class management, course management, user information management, resource category management, and test question management
	Capability evaluation module	Standard management, test content, test scheme, test management, and test archives
	Assessment management module	Assessment scheme, assessment content, examination management, and score management
	Job management module	Job release, job submission, job correction, and job evaluation
	Interactive management module	Interactive content setting, uploading interactive content, and downloading interactive content Teachers and students answer questions
	Teaching resource management module	Resource upload, resource download, resource comment, resource editing (addition, deletion, modification), and resource playback

**Table 3:** Basketball technical action test results of the experimental group and the control group before the experiment (*N* = 40).

	Early disguised dribble	Crotch dribble	Back dribble	Three-step layup	Set shot
Experience group	3.15	4.55	4.70	5.30	3.21
Control group	4.30	4.38	4.61	5.36	3.23

**Table 4:** Independent sample *t*-test of technical movement scores of students in the experimental group and the control group before the experiment.

	Experience group	Control group	*t*	*P*
Result	4.17 ± 0.96	4.39 ± 0.76	−0.35	0.727

**Table 5:** Average score of basketball technical action test of students in the experimental group and the control group after the experiment (*N* = 20).

	Early disguised dribble	Crotch dribble	Back dribble	Three-step layup	Set shot
Experience group	8.78	8.95	9.42	9.68	7.59
Control group	6.55	6.84	7.26	7.55	6.24

**Table 6:** Experimental group paired sample *t*-test of technical action test scores before and after the experiment (*N* = 20).

	Before the experiment	After the experiment	*t*	*P*
Result	4.17 ± 0.96	8.89 ± 0.83	−18.95	0.001

**Table 7:** Control group independent sample *t*-test of technical action level test scores before and after the experiment (*N* = 20).

	Before the experiment	After the experiment	*t*	*P*
Result	4.39 ± 0.76	6.88 ± 0.58	16.50	0.001

**Table 8:** Independent sample *t*-test of the average score of technical movement test in the experimental group and the control group after the experiment (*N* = 20).

	Experience group	Control group	*t*	*P*
Result	8.89 ± 0.83	6.88 ± 0.58	−4.61	0.03

## Data Availability

Some or all data, models, or codes generated or used during the study are available from the corresponding author by request.
